# Multidirectional myocardial function in bicuspid aortic valve stenosis patients: a three-dimensional speckle tracking analysis

**DOI:** 10.3389/fcvm.2024.1405754

**Published:** 2024-08-08

**Authors:** Wenhui Deng, Yuting Tan, Jiawei Shi, Shukun He, Tianshu Liu, Wenqian Wu, Yuman Li, Yali Yang, Li Zhang, Mingxing Xie, Jing Wang

**Affiliations:** ^1^ Department of Ultrasound Medicine, Union Hospital, Tongji Medical College, Huazhong University of Science and Technology, Wuhan, China; ^2^ Clinical Research Center for Medical Imaging in Hubei Province, Wuhan, China; ^3^ Hubei Province Key Laboratory of Molecular Imaging, Wuhan, China

**Keywords:** bicuspid aortic valve, left ventricle, echocardiography, myocardial function, aortic stenosis

## Abstract

**Purpose:**

The impact of aortic stenosis (AS) severity on multidirectional myocardial function in patients with bicuspid aortic valve (BAV) remains unclear, despite the recognized presence of early left ventricular longitudinal myocardial dysfunction in BAV patients with normal valve function. The aim of the study was to evaluate the multidirectional myocardial functions of BAV patients.

**Methods:**

A total of 86 BAV patients (age 46.71 ± 13.62 years, 69.4% men) with normally functioning (BAV-nf), mild AS, moderate AS, and severe AS with preserved left ventricular ejection fraction (LVEF ≥ 52%) were included. 30 healthy volunteers were recruited as the control group. Multidirectional strain and volume analysis were performed by three-dimensional speckle tracking echocardiography(3D-STE).

**Results:**

Global longitudinal strain (GLS), and global radial strain (GRS) were reduced in BAV-nf patients compared with the controls. With each categorical of AS severity from BAV-nf to severe AS, there was an associated progressive impairment of GLS and GRS (all *P* < 0.001). Global circumferential strain (GCS) did not show a significant decrease from BAV-nf to mild AS but began to decrease from moderate AS. Multiple linear regressions indicated that indexed aortic valve area (AVA/BSA), as a measure of AS severity, was an independent determinant of GLS, GCS and GRS.

**Conclusions:**

Left ventricular longitudinal myocardial reduction is observed even in patients with well-functioning bicuspid aortic valves. With each categorical increase in the grade of AS severity from normally functioning to severe aortic stenosis, there was an associated progressive impairment of longitudinal myocardial function. Furthermore, circumferential myocardial function was starting damaged from moderate AS. AVA/BSA was independently associated with multidirectional myocardial function injuries.

## 1 Introduction

BAV is the most common congenital cardiac malformation commonly associated with aortic valve disease^1^. Aortic stenosis (AS) is one of the most common valve complications in patients with BAV, and 12%–37% of BAV patients had or developed moderate to severe AS (1, 2). AS is known to significantly increase left ventricular afterload. In the presence of normal myocardial contractility, the number and diameter of myocardial fibers in the left ventricle will increase to improve the contractility of the left ventricle. As the disease progresses, heart failure occurs when the contractility is unable to balance the afterload. Actually, myocardial dysfunction and ventricular remodeling are widely demonstrated in BAV patients (3, 4). In individuals with well-functioning bicuspid aortic valves (BAV), including children, there is already evidence of reduced left ventricular longitudinal systolic function (5, 6). However, limited research has been conducted on the impact of aortic stenosis progression on multidirectional myocardial function in BAV patients.

Left ventricular systolic function serves as a crucial prognostic factor and an essential reference for clinical decision-making in patients with AS (7, 8). Similar to LVEF (9, 10), assessing myocardial systolic function accurately through a single strain measurement remains challenging (4). The clinical advantage of three-dimensional speckle tracking echocardiography (3D-STE) lies in its heightened sensitivity towards subtle functional changes (11, 12). Multidirectional analyses of longitudinal, circumferential, and radial myocardial strains can provide insights into early changes in myocardial function with increasing severity of aortic stenosis. The aims of this study were (i) to describe changes in multidirectional myocardial functions with increasing AS severity and (ii) to identify independent determinants of these functions.

## 2 Methods

### 2.1 Subjects

In this study, we initially enrolled 215 BAV patients with aortic stenosis. Following strict adherence to predefined inclusion and exclusion criteria, a total of 86 BAV patients without more than mild aortic regurgitation were ultimately included. All participants underwent comprehensive medical history review, physical examination, and transthoracic echocardiography at Wuhan Union Medical College Hospital between September 2018 and January 2021.The clinical and demographic characteristics were collected from electronic medical records or questionnaire surveys. Thirty healthy volunteers were enrolled as controls.

The inclusion criteria were as follows: (1) patients aged >18 years; (2) patients with BAV and aortic stenosis; (3) and patients had left ventricular ejection fraction (LVEF) ≥ 52% (13). The exclusion criteria were as follows: (1) moderate or severe co-existing aortic regurgitation (AR); (2) greater than moderate mitral/tricuspid regurgitation; (3) history of myocardial infarction; (4) hematologic and rheumatology diagnoses; (5) cardiomyopathy and Marfan Syndrome; (6) presence of atrial fibrillation; (7) poor image quality. Following strict adherence to these inclusion and exclusion criteria, the final population of BAV patients comprised 86 individuals (Supplementary Figure S1).

The echocardiographic subjects were divided into five groups, including controls, bicuspid aortic valve (BAV) with normally functioning valves, mild aortic stenosis (AS), moderate AS, and severe AS. The study design, data collection methods, and analysis procedures were approved by the Ethics Committee of Tongji Medical College, Huazhong University of Science and Technology (S900). Informed consent forms were signed by all participants, and all data were anonymized.

### 2.2 Conventional 2D echocardiography

A standard transthoracic echocardiography (TTE) examination was performed using the commercially available ultrasound equipment Philips EPIC 7C (Philips Medical Systems, Andover, MA, USA), following the recommendations of the American Society of Echocardiography guidelines (13). The examination included appropriate two-dimensional (2D) and Doppler imaging techniques to assess left ventricular (LV) structure, systolic and diastolic function. Left ventricular ejection fraction (LVEF) was calculated using Simpson's biplane method. Mitral inflow velocities were obtained by pulsed-wave Doppler in the apical four chamber view at early (E) and late (A) diastole stages to determine the E/A ratio. Tissue Doppler images were used to measure the average early diastolic peak velocity of both septal and lateral walls (e′). LV mass was indexed to body surface area.

The severity of aortic stenosis was classified based on the aortic peak velocity (AV), indexed aortic valve area, and mean pressure gradient (MPG) according to the guidelines recommended by the European Association of Echocardiography and American Society of Echocardiography (14). Aortic valve area (AVA) was calculated using the continuity equation, which relies on velocity time integrals obtained from both the aorta and left ventricular outflow tract. MPG was determined using the modified Bernoulli equation. Aortic valve area was adjusted for body surface area (AVA/BSA). Bicuspid aortic valve patients with normally functioning valves (BAV-nf) were defined as those with AV < 2.0 m/s, MPG < 16 mmHg (15), and absence of visual signals indicating AR in multiple views.

### 2.3 Three-Dimensional speckle tracking echocardiography (3D-STE) analysis

The 3D echocardiographic imaging was performed using a Philips X5-1 transducer (Philips Medical, Andover, Massachusetts, USA) in the apical four-chamber view. Particular attention should be paid to ensuring complete inclusion of the entire left ventricular cavity within the pyramidal dataset. Three adjacent subvolumes were captured over three consecutive cardiac cycles under a full-volume acquisition pattern. If necessary, subjects were instructed to hold their breath to minimize artifacts between subvolumes. The frame rate for 3D image acquisition ranged from 20 to 33 frames per second for patients and from 20 to 36 frames per second for controls. All images were digitally archived at the acquisition frame rate for subsequent offline analysis.

The 3D images were acquired using a commercially available software (4D LV-Analysis 3.1, TomTec Imaging Systems, Unterschleissheim, Germany). Following manual assignment of endocardial contours in each of the three apical views, the software automatically tracked 3D LV endocardial surface over time in 3D space throughout the cardiac cycles. The 3D global longitudinal strain (GLS), global circumferential strain (GCS) and global radial strain (GRS) were provided as weighted averages of the regional values of the 16 myocardial segments. The LV end-diastolic volume (LVEDV), LV end-systolic volume (LVESV), 3D left ventricular ejection fraction (3D LVEF), 3D LV mass, twist and torsion, LVEDVI (ml/m^2^ = LVEDV/BSA), LVESVI (ml/m^2^ = LVESV/BSA) were also obtained by 3D echocardiography.

### 2.4 Statistically analysis

All continuous variables are presented as mean ± standard deviation. Shapiro–Wilk test confirmed normal distribution of echocardiographic indices. In this study, we expressed categorical variables as frequencies and percentages and compared them using chi-square or exact Fisher tests. The Student's t-test was employed for comparing two groups of continuous data. One-way analysis of variance (ANOVA) was used to evaluate differences in continuous variables across subgroups of patients. *post hoc* analysis using the Bonferroni correction was used when the ANOVA *P*-value was less than 0.05. Univariate and multiple linear regression analyses identified independent demographic, clinical, and echocardiographic parameters associated with longitudinal, circumferential, and radial strain while avoiding multicollinearity between variables (tolerance set at <0.2). Due to collinearity among indicators of AS severity (MGP, AV, AVA/BSA), MGP and AV were removed from multiple linear regression analysis leaving only AVA/BSA included. Statistical analysis utilized SPSS version 19 (IBM SPSS, Chicago IL USA).

### 2.5 Repeatability analysis

Intra- and inter-observer analysis was performed to assess the values of GLS, GRS, GCS, LVEDV, and LVESV derived from 3D-STE. A total of 20 cases were randomly selected for repeatability analysis.

## 3 Results

### 3.1 Demographic characteristics

The clinical characteristics of the total BAV patients, including cardiovascular risk factors, are summarized in Table 1. A total of 86 BAV patients were included, with a mean LVEF of 63.31 ± 4.13%.

**Table 1:** Clinical and echocardiographic data of total BAV patients.

Variable	Total BAV
Number	86
Age, year	46.71 ± 13.62
Sex, male (%)	59 (68.6)
Heart rate, bpm	72.69 ± 11.08
BSA, cm/m^2^	1.73 ± 0.17
SBP, mmHg	124.57 ± 16.78
DBP, mmHg	79.03 ± 12.65
Hypertension, *n* (%)	27 (31.4)
Dyslipidaemia, *n* (%)	12 (13.9)
Smoking, *n* (%)	27 (31.4)
Normal functioning, *n* (%)	24 (27.9)
Aortic stenosis, *n* (%)	62 (72)
Mild	21 (24)
Moderate	20 (23.2)
Severe	21 (24)
LVEF (%)	63.31 ± 4.13

Data were *n* (%), mean ± SD. BSA, body surface area; SBP, systolic blood pressure; DBP, diastolic blood pressure.

### 3.2 Clinical characteristics and left ventricular function in BAV-nf patients

Table 2 outlines the clinical characteristics and echocardiographic data of BAV-nf patients and controls. BAV-nf patients exhibited higher heart rate and SBP compared to controls (*P* < 0.05). There were no significant differences in clinical features between the control group and the BAV-nf group, such as age, sex, BSA, and cardiovascular risk factors distribution (*P* > 0.05). The E/A ratio and average e′, as indicators of left ventricular diastolic function, were significantly lower in BAV-nf patients (*P* < 0.05). The AVA/BSA, MPG and AV were significantly changed in BAV-nf patients (*P* < 0.05). Compared to controls, the left ventricular volume indices (LVEDV, LVESV) were larger in BAV-nf patients (*P* < 0.05). Although there was a tendency for an increase in indexed LV mass among BAV-nf patients, this trend was not statistically significant (*P* > 0.05). In comparison to controls, both GLS (absolute value) and GRS were significantly decreased in BAV-nf patients (*P* < 0.05), while there was no significant difference observed in GCS.

**Table 2:** Comparisons between BAV patients with normally function and controls.

Variable	Control (*n* = 30)	BAV-nf (*n* = 24)	*P* value
Demographic characteristics			
Age, year	40.10 ± 13.37	37.95 ± 11.97	0.58
Sex, male (%)	18 (60)	20 (83.3)	0.07
Heart rate, bpm	70.50 ± 9.87	78.33 ± 13.29	0.02
BSA, m^2^	1.72 ± 0.18	1.77 ± 0.18	0.31
SBP, mmHg	117.00 ± 9.42	124.08 ± 15.77	0.04
DBP, mmHg	74.83 ± 8.00	80.21 ± 12.99	0.07
Hypertension, *n* (%)	3 (10)	3 (12.5)	–
Dyslipidaemia, *n* (%)	0	3 (12.5)	–
Smoking, *n* (%)	8 (26.7)	7 (29.2)	0.19
Conventional echocardiographic characteristics			
E-wave, cm/s	0.89 ± 0.24	0.85 ± 0.18	0.41
A-wave, cm/s	0.66 ± 0.21	0.75 ± 0.24	0.18
E/A ratio	1.43 ± 0.47	1.18 ± 0.24	0.03
Average e′, cm/s	0.12 ± 0.02	0.11 ± 0.02	0.01
E/e′ ratio	7.63 ± 2.14	8.36 ± 2.73	0.27
AVA/BSA, cm^2^/m^2^	1.65 ± 0.37	1.39 ± 0.31	0.009
MPG, mmHg	3.38 ± 0.93	4.63 ± 1.98	0.003
AV, m/s	1.22 ± 0.15	1.39 ± 0.31	0.01
LV volume and multidirectional myocardial function			
LVEDV, ml	83.25 ± 11.14	95.70 ± 12.31	0.002
LVESV, ml	24.79 ± 5.19	29.28 ± 5.23	0.005
LVEF, %	69.89 ± 5.11	69.34 ± 5.37	0.08
Indexed LV mass, g/m^2^	69.76 ± 8.67	73.38 ± 8.71	0.15
Global longitudinal strain, %	−25.91 ± 2.36	−20.53 ± 1.12	<0.001
Global circumferential strain, %	−38.36 ± 1.32	−37.12 ± 2.23	0.62
Global radial strain, %	51.41 ± 2.11	49.26 ± 3.27	0.001
Twist, °	11.32 ± 5.29	11.15 ± 5.11	0.93
Torsion, °	1.54 ± 0.66	1.53 ± 0.92	0.98

Data were *n* (%), mean ± SD. BAV-nf, bicuspid aortic valve with normally functioning; BSA, body surface area; SBP, systolic blood pressure; DBP, diastolic blood pressure; E-wave, peak early diastolic velocity of atrial filling; A-wave, peak late diastolic velocity of atrial filling; E/A ratio, ratio of early-to-late diastolic mitral peak; Average e’, the average of TDI-derived early diastolic peak velocity of the septal and lateral wall; E/e′ = ratio of E velocity to e’; MPG, mean pressure gradient; AV, aortic peak velocity; LVEDV, left ventricular end-diastolic volume; LVESV, left ventricular end-systolic volume; LVEF, left ventricular ejection fraction. *P* value less than 0.05 was considered statistically significant.

### 3.3 Changes of left ventricular function with increasing aortic stenosis severity

Table 3 outlines the clinical characteristics and left ventricular function of BAV according to severity of AS (BAV-nf, mild AS, moderate AS, severe AS). Patients with moderate and severe AS were older than those with mild AS and BAV-nf (*P* < 0.001). The distribution of males with moderate AS was lower than that of other groups (*P* < 0.001). BSA in the severe AS was smaller than that in mild AS (*P* = 0.007). The incidence of hypertension history in BAV patients with AS was significantly higher than that in patients without AS (*P* = 0.03). There were no significant differences in heart rate, SBP, DBP, or smoking among the groups (*P* > 0.05). The average e′ and 3D LVEF decreased in patients with moderate and severe AS compared to those with mild AS or without AS (*P* < 0.001). No significant change in LVEDV was observed with increasing severity of AS (*P* > 0.001). Patients with moderate and severe AS showed an increase in LVESV compared to those with mild AS or without AS (*P* < 0.001).

**Table 3:** Clinical characteristics and echocardiographic data of BAV patients according to severity of aortic stenosis.

Variable	BAV-nf (*n* = 24)	Mild AS (*n* = 20)	Moderate AS (*n* = 20)	Severe AS (*n* = 21)	*P value
Demographic characteristics					
Age, year	37.95 ± 11.97	42.80 ± 12.19	53.75 ± 7.68^#^	53.71 ± 14.14	<0.001
Sex, male (%)	20 (83.3)	19 (95)	7 (35)	13 (61.9)	<0.001
Heart rate, bpm	78.33 ± 13.29	69.14 ± 7.31	70.60 ± 10.76	71.09 ± 9.88	0.29
BSA, m^2^	1.77 ± 0.18	1.81 ± 0.14	1.69 ± 0.13	1.65 ± 0.18	0.007
SBP, mmHg	124.08 ± 15.77	124.50 ± 18.03	126.60 ± 13.31	123.62 ± 20.08	0.96
DBP, mmHg	80.21 ± 12.99	81.85 ± 13.76	77.80 ± 11.57	76.19 ± 12.29	0.49
Hypertension, *n* (%)	3 (12.5)	6 (28.6)	8 (40)	10 (47.6)	0.03
Dyslipidaemia, *n* (%)	3 (12.5)	2 (10)	5 (25)	3 (14.3)	–
Smoking, *n* (%)	7 (29.2)	10 (50)	6 (30)	4 (19)	0.19
Conventional echocardiographic characteristics					
E-wave, cm/s	0.85 ± 0.18	0.80 ± 0.22	0.79 ± 0.19	0.86 ± 0.23	0.60
A-wave, cm/s	0.75 ± 0.24	0.69 ± 0.20	0.79 ± 0.18	0.84 ± 0.30	0.28
E/A ratio	1.18 ± 0.24	1.25 ± 0.53	1.04 ± 0.33	1.17 ± 0.54	0.46
Average e′, cm/s	0.11 ± 0.02	0.10 ± 0.02	0.08 ± 0.01^#^	0.07 ± 0.01	<0.001
E/e′ ratio	8.36 ± 2.73	8.51 ± 3.48	10.39 ± 3.20	13.05 ± 3.74	<0.001
AVA/BSA, cm^2^/m^2^	1.39 ± 0.31	1.08 ± 0.14^#^	0.71 ± 0.08^#^	0.48 ± 0.06^#^	<0.001
MPG, mmHg	4.63 ± 1.98	14.94 ± 2.19^#^	28.74 ± 8.41^#^	55.67 ± 15.60^#^	<0.001
AV, m/s	1.39 ± 0.31	2.67 ± 0.12^#^	3.55 ± 0.22^#^	4.76 ± 0.57^#^	<0.001
LV volume and multidirectional myocardial function					
LVEDV, ml	95.70 ± 12.31	110.18 ± 16.23	98.64 ± 42.43	120.66 ± 62.37	0.19
LVESV, ml	29.28 ± 5.23	33.16 ± 7.35	42.44 ± 20.29^#^	51.73 ± 28.48	<0.001
LVEF, %	69.34 ± 5.37	68.18 ± 3.56	59.10 ± 4.69^#^	56.63 ± 2.90	<0.001
Indexed LV mass, g/m^2^	73.38 ± 8.71	83.89 ± 13.23	93.29 ± 37.77	114.55 ± 47.18	<0.001
Global longitudinal strain, %	−20.53 ± 1.12	−19.05 ± 1.02^#^	−18.63 ± 1.06^#^	−15.63 ± 2.30^#^	<0.001
Global circumferential strain, %	−37.12 ± 2.23	−36.46 ± 2.40	−30.17 ± 3.09^#^	−28.32 ± 1.57	<0.001
Global radial strain, %	49.26 ± 3.27	47.36 ± 1.54^#^	39.88 ± 2.87^#^	35.57 ± 3.02^#^	<0.001
Twist, °	11.15 ± 5.11	16.50 ± 7.59	15.25 ± 8.29	17.22 ± 9.03	0.19
Torsion, °	1.53 ± 0.92	2.07 ± 0.95	2.06 ± 1.11	2.06 ± 1.05	0.23

Data were *n* (%), mean ± SD. AS, aortic stenosis; BSA, body surface area; SBP, systolic blood pressure; DBP, diastolic blood pressure; E-wave, peak early diastolic velocity of atrial filling; A-wave, peak late diastolic velocity of atrial filling; E/A ratio, ratio of early-to-late diastolic mitral peak; Average e’, the average of TDI-derived early diastolic peak velocity of the septal and lateral wall; E/e′ = ratio of E velocity to e’; AVA/BSA, aortic valve area normalized to body surface area; MPG, mean pressure gradient; AV, aortic peak velocity; LVEDV, left ventricular end-diastolic volume; LVESV, left ventricular end-systolic volume; LVEF, left ventricular ejection fraction.

* *P***-**value, calculated by ANOVA comparing the means of the variables for the four groups. *Post hoc* analysis using the Bonferroni correction was used when the ANOVA *P*-value was <0.05.

^#^ *P* < 0.05 vs. preceding AS category with Bonferroni correction.

**Figure 1:**
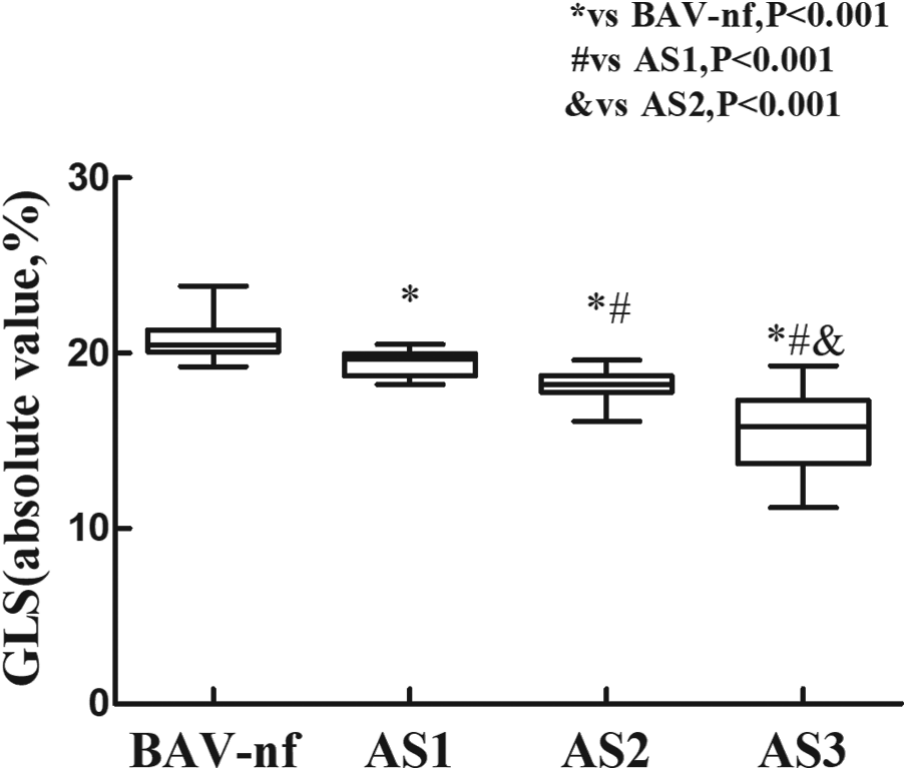
Impairment of left ventricular global longitudinal strain with increasing aortic stenosis severity (*P* < 0.001 by ANOVA). With each categorical increase in aortic stenosis severity grade from normally functioning to severe aortic stenosis, there was an associated progressive impairment of global longitudinal strain (all *P* < 0.05 by Bonferroni correction).

**Figure 2:**
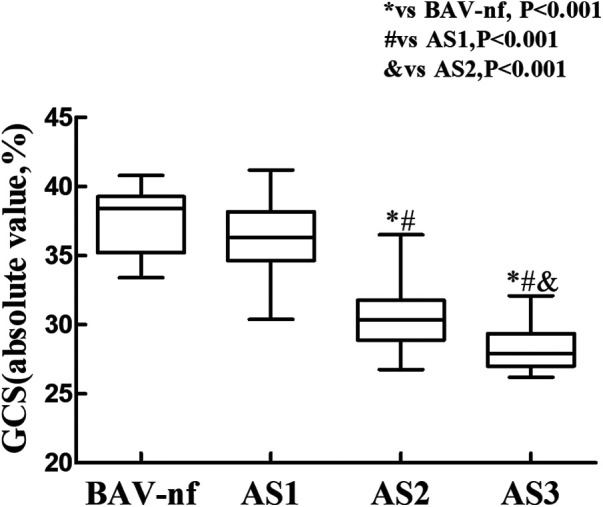
Impairment of left ventricular global circumferential strain with increasing aortic stenosis severity (*P* < 0.001 by ANOVA). There was no significant difference in left ventricular global circumferential strain between normally functioning and mild stenosis, and moderate between severe stenosis (all *P* > 0.05 by Bonferroni correction). But global circumferential strain progressively worsened from mild to moderate (*P* < 0.05).

**Figure 3:**
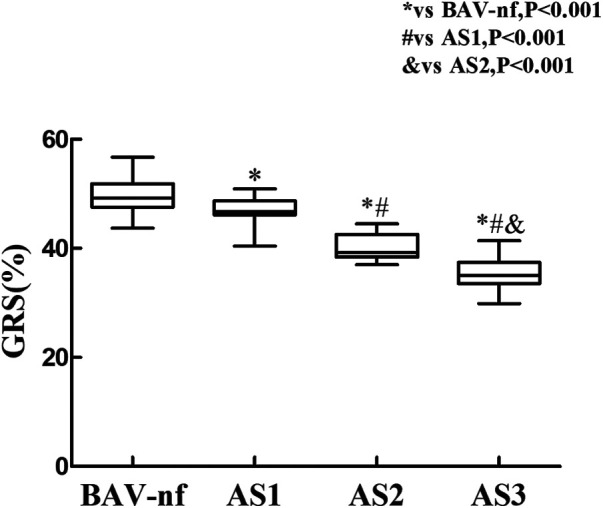
Impairment of left ventricular global radial strain with increasing aortic stenosis severity (*P* < 0.001 by ANOVA). With each categorical increase in aortic stenosis severity grade from normally functioning to severe aortic stenosis, there was an associated progressive impairment of global radial strain (all *P* < 0.05 by Bonferroni correction).

The one-way ANOVA revealed a significant decrease in GLS and GRS as the severity of AS increased (*P* < 0.001). *post hoc* analysis demonstrated that there was a decrease in GLS and GRS with each categorical increase in the grade of AS severity, from BAV-nf to severe AS (*P* < 0.001). One-way ANOVA showed the GCS declined with increasing AS severity (*P* < 0.001). *Post hoc* analysis showed that there were no significant differences in GCS between BAV-nf and mild AS, and between moderate AS and severe AS. There were no significant differences in LV twist and torsion with increasing AS severity (*P* > 0.05). Figures 1–3 demonstrate a progressive impairment in GLS (absolute value), GCS (absolute value), and GRS with increasing AS severity.

### 3.4 Independent factors associated with myocardial function

Table 4 summarizes the independent factors associated with GLS, GCS, GRS in total BAV patients. Factors that may affect myocardial strains were included in the univariate regression analysis. The AV and MPG were not included in the multivariate regression analysis models due to significant collinearity with SBP and AVA/BSA in our study. Results showed that the AVA/BSA was independently associated with an impaired GLS (*β* = −9.45, *P* < 0.001), GCS (*β* = −8.54, *P* < 0.001), GRS (*β* = 12.01, *P* < 0.001), even after correcting for age, sex, heart rate, BSA, SBP, DBP, hypertension, dyslipidemia, smoking, indexed LV mass.

**Table 4:** Univariate and multivariate linear regression analysis for multidirectional myocardial functions in BAV patients.

Variable	Global longitudinal strain				Global circumferential strain (%)				Global radial strain (%)			
	Univariate		Multivariate		Univariate		Multivariate		Univariate		Multivariate	
	*β*	*P*	*β*	*P*	*β*	*P*	*β*	*P*	*β*	*P*	*β*	*P*
Age, year	0.14	<0.001	0.02	0.22	0.17	<0.001	0.02	0.57	−0.25	<0.001	−0.02	0.48
Sex, (female = 0, male = 1)	−1.75	0.03	0.69	0.20	−3.51	0.01	−0.87	0.33	4.63	0.002	−0.16	0.86
Heart rate, bpm	−0.06	0.047	−0.02	0.23	−0.10	0.02	−0.03	0.34	0.15	0.02	0.03	0.26
BSA, m^2^	−0.09	0.01	−1.42	0.30	−9.42	0.01	−2.61	0.25	14.17	<0.001	5.46	0.02
SBP, mmHg	−0.004	0.85			−0.001	0.96			0.002	0.96		
DBP, mmHg	−0.03	0.36			−0.07	0.07			0.08	0.15		
Hypertension	2.24	0.004	0.21	0.63	3.00	0.004	−0.53	0.47	−4.59	0.002	0.51	0.51
Dyslipidaemia	−0.02	0.98			−0.44	0.76			0.06	0.97		
Smoking	−0.20	0.81			−0.83	0.45			0.96	0.52		
Indexed LV mass, g/m^2^	0.04	<0.001	0.01	0.03	0.05	<0.001	0.18	0.11	−0.08	<0.001	−0.02	0.04
AVA/BSA, cm^2^/m^2^	−9.56	<0.001	−9.45	<0.001	−9.97	<0.001	−8.54	<0.001	13.77	<0.001	12.01	<0.001

BSA, body surface area; SBP, systolic blood pressure; DBP, diastolic blood pressure; AVA/BSA, aortic valve area normalized to body surface area. *P* value less than 0.05 was considered statistically significant.

### 3.5 Repeatability analysis

The intra- and inter-observer agreement of GLS, GRS, GCS, LVEDV, LVESV values was analyzed using the interclass correlation coefficient (ICC). We randomly selected 20 cases for repeatability analysis. The intraclass correlation coefficients for intra-and inter-observer variability were 0.97 [95% confidence interval (CI) 0.94–0.99] and 0.98 (95% CI 0.95–0.99) for GLS, 0.91 (95% CI 0.79–0.96) and 0.93 (95% CI 0.84–0.97) for GCS, 0.88 (95% CI 0.73–0.95) and 0.84 (95% CI 0.64–0.93) for GRS, 0.98 (95% CI 0.95–0.99) and 0.95 (95% CI 0.88–0.98) for LVEDV, 0.97 (95% CI 0.92–0.99) and 0.97 (95% CI 0.91–0.99) for LVESV, respectively.

## 4 Discussion

BAV is a genetically heterogeneous disease, and AS is one of its most common complications (1). In general population, AS is an important risk factor for cardiovascular morbidity and mortality (16). It causes left ventricular remodeling with hypertrophy and myocardial function impairment with the chronic pressure overload (17, 18). Previous studies have shown that BAV patients, even with normally functioning, have lower ventricular longitudinal contraction compared with normal population. However, there is limited data on the effects of development and progression of AS on multidirectional myocardial function in BAV patients.

### 4.1 Left ventricular systolic and diastolic function in early stage

In our study, patients with bicuspid aortic valve (BAV) and normal cardiac function exhibited significantly lower global longitudinal strain (GLS) and global radial strain (GRS), while no significant difference in ejection fraction (EF) was observed between BAV-nf group and controls. As the primary indicators of diastolic function, the E/A ratio and average e′ (19) were lower in both group but still within the normal range. It suggests that diastolic function might be lower in BAV patients with normal EF and normally functioning, which is in agreement with the reported results (3, 20). It is a known fact that decreased aortic elasticity and increased stiffness are the most prominent features of the aorta in patients with BAV. Some studies have shown that aortic stiffening is associated with left ventricular dysfunction (21, 22), while others have shown weak or no association between aortic stiffening and left ventricular dysfunction (3, 5). Studies have demonstrated that the aortic valve area and abnormal aortic flow patterns impact LV remodeling (4, 23). Abnormal aortic flow patterns are closely related to aortic valve area (23), but can increase ventricular afterload even without significant AS (24). In our study, the aortic valve area index (AVA/BSA) and hemodynamic parameters (AV, MPG) were within the normal reference range in BAV-nf patients but significantly different from controls (*P* < 0.05). The AVA/BSA was independently associated with GLS and GRS. Thus, abnormal aortic flow patterns and aortic stiffening can theoretically increase ventricular afterload which may explain early reduced left ventricular myocardial strain in BAV patients. Furthermore, we speculate that reduced AVA at an early stage of BAV may be indicative of early left ventricular remodeling progression.

### 4.2 The effects of development of AS on multidirectional myocardial function

In contrast to previous studies (25, 26) demonstrating a reduced global longitudinal strain (GLS) and an increased global circumferential strain (GCS) in TAV-AS, our study revealed no significant alteration in GCS from normal to mild stenosis but exhibited a decline starting from moderate AS. We speculate that the reasons may be as follows: under normal myocardial contraction force conditions, the middle circumferential myocardium can enhance myocardial contractility to compensate for the decreased motion of longitudinal and radial myocardium (18, 26). However, myocardial contractility was reduced in the early stage of BAV patients with normal valve function. The middle circumferential myocardium may compensate by maintaining myocardial movement and/or myocardial thickening, which could explain why GCS is not elevated in BAV patients with mild AS. When left ventricular hypertrophy cannot adapt to increased afterload, “afterload mismatch” occurs, resulting eventually in a reduction of LVEF (27). This may explain why LVEF and LVESV showed statistically significant changes from moderate AS and were consistent with the trend of GCS. However, a decrease in LVEF indicates poor prognosis for patients (28, 29). Torsion and twist of the left ventricle are lower in BAV patients with severe AS compared to those in the control group, suggesting impaired left ventricular systolic function and mechanical efficiency. Therefore, multiple deformation indices of left ventricular myocardium function are essential for understanding the pathophysiologic mechanics of LV dysfunction.

Univariate regression analysis showed that AVA/BSA was correlated with GLS, GCS, and GRS in patients with BAV-AS. Despite correcting for confounding risks on multivariable analysis, AVA/BSA remained independently associated with left ventricular strains, suggesting that the degree of stenosis is an important factor for multidirectional myocardial functions. Additionally, age and LVMI were also found to be associated with myocardial injury. Age-related myocardial injury suggests a possible atherosclerotic mechanism (30). Increased left ventricular mass is associated with a higher risk of cardiovascular adverse events (31).

Little data is available on the multidirectional myocardial functions in the progression of AS, although early left ventricular longitudinal myocardial dysfunction is known to be present in BAV patients with normal functioning. 3D-STE imaging technology can quantitatively assess the multidirectional myocardial function of the left ventricle and has been extensively recognized for its early detection of myocardial injury (12). In our study, left ventricular myocardial injury began with impairment of longitudinal fibers. This may be attributed to the fact that subendocardial longitudinal fibers are more sensitive to increased wall stress and reduced myocardial perfusion (32). Unlike strains in other directions, GRS refers to changes in wall thickness along a vertical line from the endocardium to the epicardium boundary at a specific point. In fact, GRS may not be a meaningful indicator in clinical practice as it does not refer to a certain level of muscle fiber function but exhibits great variability (18, 33). Studies have demonstrated an association between reduced left ventricular longitudinal myocardial function and myocardial fibrosis (34, 35). As the disease progresses, changes in AS-induced myocardial fibrosis begin in the subendocardium and gradually progress towards transmural involvement (36). Studies have indicated that GLS can provide improved risk stratification for severe AS and may influence optimal timing for AVR (37). Earlier detection of subclinical myocardial dysfunction by speckle tracking echocardiography may allow earlier identification of patients at risk for irreversible myocardial damage.

### 4.3 Limitations

The present study employed a cross-sectional design, with data collected from a single-center and a relatively small number of patients. The sample size was limited due to stringent inclusion criteria. Due to the limited sample size, we were unable to conduct a comparative analysis between symptomatic and asymptomatic patients with severe BAV-AS. Recently, left atrial function has emerged as a novel and early indicator of left ventricular dysfunction. However, in this study, we did not specifically evaluate the performance of left atrial function. Moreover, the comparison between BAV and tricuspid aortic valve in the context of aortic stenosis (AS) was beyond the scope of this study. Additionally, the long-term prognostic implications of multidirectional myocardial strain remain unknown due to lack of follow-up studies; therefore, future large-scale longitudinal studies are warranted.

## 5 Conclusions

Patients with BAV exhibit early myocardial dysfunction in the progression of stenosis, despite having a normal LVEF. Left ventricular longitudinal myocardial reduction occurs even in BAV patients with well-functioning aortic valves. With each categorical increase in the severity grade of AS, ranging from normally functioning to severe aortic stenosis, there is a corresponding progressive impairment of longitudinal myocardial function. Furthermore, circumferential myocardial function begins to deteriorate from moderate AS onwards. The AVA/BSA ratio serves as an independent indicator for assessing the severity of AS and its association with multidirectional myocardial functional impairments.

## Data Availability

The raw data supporting the conclusions of this article will be made available by the authors, without undue reservation.
