# Supplementary material for: Multidirectional myocardial function in bicuspid aortic valve stenosis patients: a three-dimensional speckle tracking analysis
Source: Front Cardiovasc Med. 2024 Aug 8;11:1405754. doi: 10.3389/fcvm.2024.1405754 (PMC11338759; doi:10.3389/fcvm.2024.1405754)
Supplement: Supplementary file 1 [file Presentation1.pptx]

## Slide 1
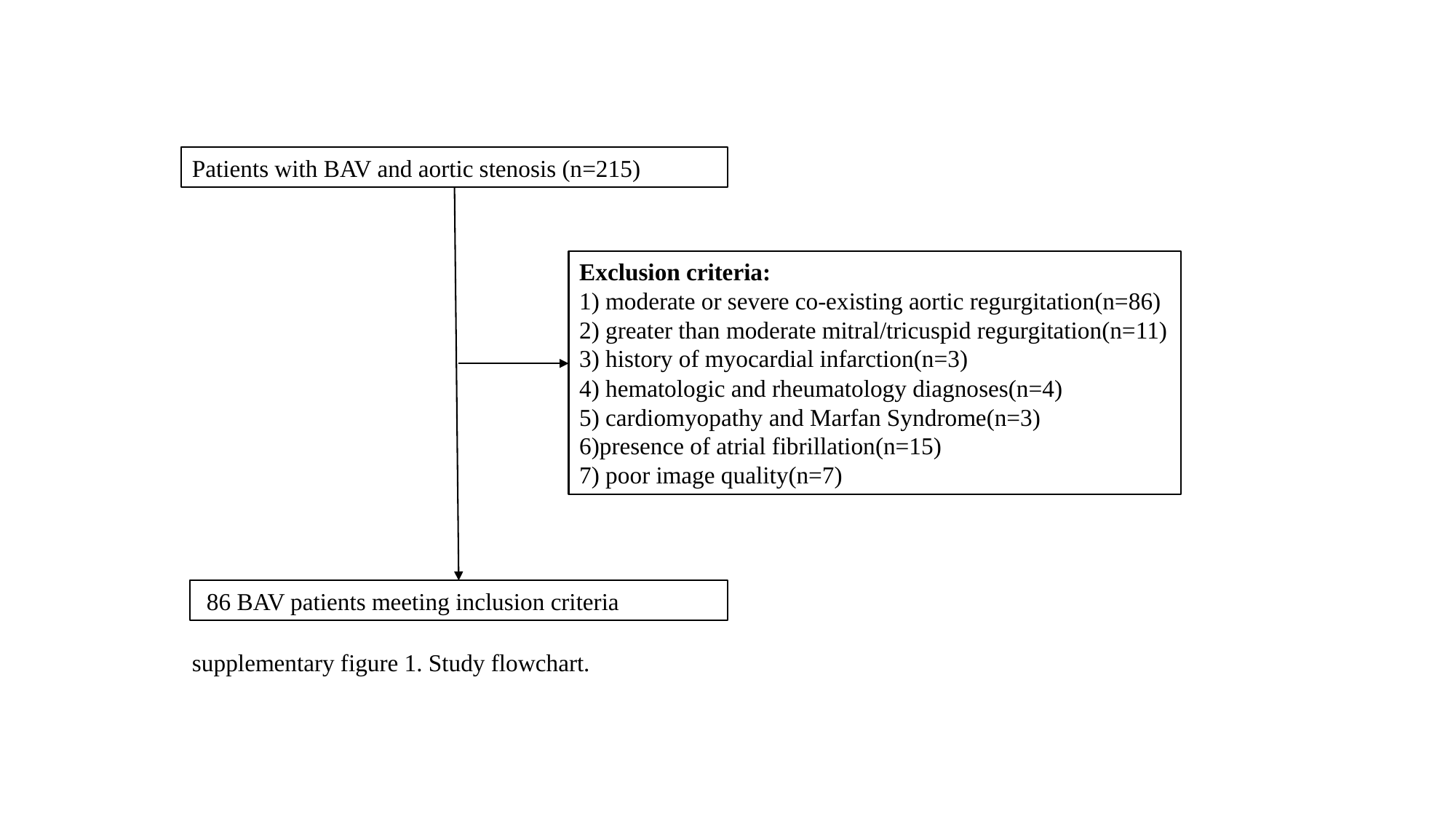

Patients with BAV and aortic stenosis (n=215)
Exclusion criteria:
1) moderate or severe co-existing aortic regurgitation(n=86)
2) greater than moderate mitral/tricuspid regurgitation(n=11)
3) history of myocardial infarction(n=3)
4) hematologic and rheumatology diagnoses(n=4)
5) cardiomyopathy and Marfan Syndrome(n=3)
6)presence of atrial fibrillation(n=15)
7) poor image quality(n=7)
 86 BAV patients meeting inclusion criteria
supplementary figure 1. Study flowchart.
